# HIV self-testing: South African young adults’ recommendations for ease of use, test kit contents, accessibility, and supportive resources

**DOI:** 10.1186/s12889-019-6402-4

**Published:** 2019-01-29

**Authors:** Tiarney D. Ritchwood, Amanda Selin, Audrey Pettifor, Sheri A. Lippman, Hailey Gilmore, Linda Kimaru, Jennifer Hove, Ryan Wagner, Rhian Twine, Kathleen Kahn

**Affiliations:** 1 Department of Community and Family Medicine, Duke University, 2200 W Main St, Durham, NC 27705 USA; 2 Carolina Population Center, University of North Carolina, Chapel Hill, USA; 3 MRC/Wits Rural Public Health and Health Transitions Research Unit (Agincourt), School of Public Health, Faculty of Health Sciences, University of the Witwatersrand, Johannesburg, South Africa; 4 Department of Epidemiology, Gillings School of Global Public Health, University of North Carolina at Chapel Hill, Chapel Hill, USA; 5 Department of Medicine, University of California, San Francisco, USA

**Keywords:** HIV self-testing, Young adults, South Africa, Rural, Recommendations

## Abstract

**Background:**

The uptake of HIV self-testing (HIVST) could address socio-structural barriers that prevent South African youth from utilizing the testing resources available in their communities. However, to facilitate this, we must tailor components of the HIVST kit and process to ensure that we reach and encourage youth to test. The purpose of this study to elucidate concerns and issues regarding HIVST rollout among South African youth.

**Methods:**

This study was conducted in two phases: 1) focus group discussions with rural, South African youth aged 18–24 and 2) direct observations of participants completing with an oral HIVST kit and/or a blood based HIVST kit. In phase 2a participants were invited to try both an oral and blood-based HIVST kit. In phase 2b, participants selected the HIVST kit of their choice.

**Results:**

We enrolled 35 unique participants in phase 1, 20 participants in phase 2a, and 40 participants in phase 2b. While the focus group discussions highlighted hypothetical HIVST use only, participants appreciated the privacy that the HIVST could afford them. However, they expressed concerns about whether HIVST could be trusted due to false positives and negatives, as well as whether a person would be able to emotionally handle the results if they tested alone. They suggested that the kits be used alongside someone who could provide support. In phases 2a and 2b, participants were overwhelmingly positive about both kits regarding ease of use and whether their results could be trusted. The participants, however, experienced more challenges with the blood-based versus oral test. When given the choice in phase 2b, most participants (80%) chose the oral HIVST over the blood-based HIVST.

**Conclusions:**

During the focus group discussions, participants raised concerns about the validity of HIVST, lack of emotional support when testing alone, and the cost of HIVST kits, all of which could be addressed through current testing campaigns. Most of those who actually tested had positive experiences with HIVST and would recommend it to their friends. When offered a choice, most preferred the oral test.

**Trial registration:**

NCT03162965, registered 19th May 2017.

**Electronic supplementary material:**

The online version of this article (10.1186/s12889-019-6402-4) contains supplementary material, which is available to authorized users.

## Background

South Africa continues to be disproportionately burdened by the HIV pandemic, with over 6.4 million individuals currently living with the virus [1]. In 2010, the South African government launched a large-scale, HIV testing campaign that resulted in a reported increase in annual HIV testing from 50% in 2008 to 66.5% in 2014 [2]. Despite this successful outcome, there are a significant number of people who have not tested recently [2], demonstrating that current efforts to reach UNAIDS and country targets to reduce transmission rates are insufficient. Moreover, the proportion of adolescents accessing HIV testing services has been low, with youth (15–24 years) being less likely to test than adults (25–49 years), indicating that many at high risk for infection are unaware of their HIV status [1].

Lack of awareness of one’s HIV status drives forward HIV transmission and leads to treatment delays [3, 4]. It is, therefore, critical that we improve testing uptake and reduce the number of undiagnosed cases of HIV. To do this, we must expand delivery options that have the potential to overcome current barriers to HIV testing, including stigma, discrimination, and breach of confidentiality [5]. HIV self-testing (HIVST) is one such option that may increase both HIV testing uptake and frequency, as well as improve early diagnosis, all of which are linked to decreased HIV-related morbidity, mortality, and transmission [6–10].

HIVST kits, which are available for use, sale and distribution in a number of Western countries, as well as in some parts of Africa (i.e., Kenya, South Africa, Nigeria) and Brazil, enable individuals to perform a rapid HIV antibody test on their own [11]. This could address socio-structural barriers associated with testing, including concerns regarding confidentiality and healthcare access [12, 13]. Additionally, HIVST may ameliorate stigma around HIV testing, a formidable barrier [13]. While previous research in South Africa and elsewhere have demonstrated the acceptability of HIVST among South African adults, more youth-focused research is needed to develop a better understanding of the nuances of HIVST that could impact uptake [7, 14–16]. This information is critical, as it sheds light on how strategies aimed at linking youth to care will work outside a clinic setting, allowing us to implement and replicate the results of successful trials. In this study, we aimed to elucidate concerns and issues regarding HIVST rollout, including acceptability of HIVST among South African youth; usability of HIVST kits; preference for blood or oral-based HIVST kits; and recommendations that would help to facilitate HIVST use in their communities. This information was used to inform a randomized trial [17].

## Methods

We conducted formative research to inform the Testing Innovations study, a randomized trial designed to assess whether offering HIVST would increase testing uptake among young women and their peers and partners in the Agincourt Health and socio-Demographic Surveillance Site (HDSS) in rural Mpumalanga Province. The study was conducted in two phases in late 2015 and early 2016: 1) formative focus groups and 2) direct observations of participants. Regarding our phase 1 focus groups, we sought to determine whether HIVST was acceptable to members of the target population. We conducted four semi-structured, focus groups that were comprised of serostatus unknown men and women between the ages of 18 and 24. Two experienced fieldworkers led the focus group discussions using an interview guide developed by the study team (See Additional file 1). For phase 2, we conducted direct observations of self-reported HIV-negative men and women, ages 18–24, completing both the saliva/oral and blood test (phase 2a) or selecting which testing they would prefer to take, either the saliva or the blood test (phase 2b) to better understand any challenges with self-testing and the associated materials. Convenience sampling was used in phases 1 and 2 to recruit participants from venues in the community frequented by youth in the target demographic.

In phase 2a, we recruited local youth to use the HIVST kits in the presence of a trained counselor in a private room. We asked the participants to try two different self-testing kits, a blood-based test and oral HIV test. Participants were given a set of written instructions that provided step-by-step guidance on how to use the test kits in English and Shangaan. HIV counselors first showed the participants how to use the HIVST kits correctly by following the instructions provided but did not actually complete a self-test. Participants then conducted HIVST independently, with counselors observing and documenting whether the participants followed the instructions using a standardized observation checklist. At the conclusion of the HIVST, counselors confirmed the participant’s interpretation of the results. Participants then responded to a brief questionnaire administered via Computer Assisted Personal Interview (CAPI) that inquired about their experiences using the test kits (e.g. clarity of instructions, comfort using the test, confidence in test result, difficulty performing the test and reading results). In phase 2b, procedures mirrored those of phase 2a with one exception; the participants only completed one HIVST of their choice, either the blood-based test or the oral test. Participants in phases 2a and 2b did not overlap. Participants were referred to a local clinic for confirmatory testing.

### Data analysis

Focus group discussions were translated and transcribed and imported into Atlas.ti, version 7 for data analysis. We used a qualitative content analytic approach to analyze transcripts, inductively [18]. To create the coding manual, we compared the interview transcripts and then categorized common responses. A single analyst coded the transcripts using agreed upon codes and created data reduction tables to identify themes and sub-themes related to the structural codes, noting the frequency of those themes and sub-themes, and highlighting illustrative quotes. Ultimately, themes were developed based on patterns and topics that persisted throughout the interviews. We used SAS 9.4 to perform descriptive analysis of questionnaire data. We calculated means or medians for continuous variables and frequencies for categorical variables.

## Results

### Participants

#### Phase 1: Focus group discussions

Tables 1 and 2 summarize the participants’ demographic characteristics. In phase 1, there were 35 participants, 19 young men (*median*_age_ = 22, interquartile range: 4) and 16 young women (*median*_age_ = 18.5, interquartile range: 3). There were 8 participants in each female focus group and 9–10 participants in each male focus group.

**Table 1:** Participant demographics

	Phase 1 (%)	Phase 2a (%)	Phase 2b (%)
Gender			
Male	19	10	20
Female	16	10	20
Age (Median, interquartile range)	19, 4	21, 2	21, 2
Highest level of education			
Primary	–	1 (5)	1 (3)
Secondary	–	7 (35)	14 (35)
Matric	–	10 (50)	14 (35)
Tertiary	–	2 (10)	11 (28)
Paid work in the past 6 months?			
Yes	–	3 (15)	4 (10)
No	–	17 (85)	36 (90)
When was the last time you were tested for HIV before today?			
Within the last 6 months	–	13 (65)	13 (33)
6–12 months	–	1 (5)	11 (28)
> 1–2 years	–	3 (15)	7 (18)
> 2–5 years	–	1 (5)	2 (5)
I never tested for HIV before today	–	2 (10)	7 (18)
Where did you last test for HIV?			
At the clinic	–	14 (77)	20 (61)
At an NGO	–	4 (22)	8 (24)
At a private doctor	–	0	1 (3)
As part of a study	–	0	4 (12)

Note: Percentages exceed 100% due to rounding; Two people did not provide responses to the last question in phase 2a

**Table 2:** Participant HIVST Kit Experiences

	Phase 2a		Phase 2b	
	Oral fluid *n* = 20 (%)	Finger stick *n* = 20(%)	Oral fluid *n* = 32(%)	Finger stick *n* = 8(%)
Did you complete the HIV test today?				
Yes	20 (100)	20 (100)	31 (97)	8 (100)
No	–	–	1 (3)	–
Were you able to collect the sample?				
Yes	19 (95)	20 (100)	–	–
No	1 (5)	–	–	–
How comfortable did you feel collecting your own sample with the test kit?				
Very comfortable	16 (84)	14 (70)	24 (77)	6 (75)
Somewhat comfortable	3 (16)	4 (20)	6 (19)	2 (25)
Somewhat uncomfortable	–	2 (10)	1 (3)	–
How much pain did you feel when you collected your sample with the test kit?				
No pain	19 (100)	17 (85)	31 (100)	8 (100)
Not a lot of pain	–	1 (5)	–	–
A little pain	–	2 (10)	–	–
How easy or difficult was it to collect your sample with the test?				
Very easy	18 (95)	12 (60)	29 (94)	7 (88)
Somewhat easy	1 (5)	5 (25)	2 (6)	1 (13)
Somewhat difficult	–	3 (15)	–	–
Most difficult part				
Too many steps/too complicated	–	1	–	–
Have a hard time with blood	–	2	–	–
How easy or difficult was it to conduct the test once you had collected the sample?				
Very easy	18 (95)	13 (65)	27 (87)	7 (88)
Somewhat easy	1 (5)	7 (45)	4 (13)	1 (13)
How easy or difficult was it to interpret the test results – in other words – to know if the test was positive or negative?				
Very easy	18 (95)	18 (90)	31 (100)	8 (100)
Somewhat difficult	1 (5)	2 (10)	–	–
Do you trust the test result, meaning do you believe the result?				
Yes	19 (100)	20 (100)	31 (100)	8 (100)
How confident are you that you used the test correctly?				
Very confident	17 (89)	11 (55)	29 (94)	7 (88)
Somewhat confident	2 (11)	5 (25)	2 (6)	1 (13)
Somewhat unconfident	–	4 (20)	–	–
How confident are you that you could use the test correctly in the future on your own?				
Very confident	19 (95)	20 (100)	31 (100)	8 (100)
Somewhat confident	1 (5)	–	–	–
Take a moment to think about when you used the HIV test. What did you like most about the test? (Please pick your top answer)				
Trusted that results were accurate	–	3 (15)	1 (3)	–
That the test was easy to use	4 (20)	5 (25)	8 (26)	1 (13)
That the test was comfortable to use	2 (10)	1 (5)	1 (3)	1 (13)
That I could do the test myself	14 (70)	10 (50)	19 (61)	5 (63)
It was a new way to test for HIV	–	1 (5)	1 (3)	1 (13)
It used saliva to test for HIV	–	–	1 (3)	–
What did you like least about the test?				
Fear of Pricking	–	3 (15)	–	–
Test was Painful	–	–	–	1 (13)
Waiting Time	–	–	–	1 (13)
There was nothing I disliked about the test	20 (100)	17 (85)	31 (100)	6 (75)
For your next HIV test, would you prefer to go to the clinic or test yourself at home?				
Health Worker	–	–	8 (20)	–
Do it yourself at home	20 (100)	–	32 (80)	–
If you would prefer to test yourself at home, would you like someone to be there with you?				
Yes	11 (55)	–	6 (19)	–
No	9 (45)	–	26 (81)	–
If you would like to have someone with you, who would you like to be there?				
A friend	–	–	1 (17)	–
A family member	–	–	4 (67)	–
A sexual partner	–	–	1 (17)	–
If HIV self-test kits were available (like the one you used today), how likely is it that you would use one to test yourself for HIV?				
Very likely	18 (90)	–	40 (100)	–
Somewhat likely	1 (5)	–	–	–
Somewhat unlikely	1 (5)	–	–	–
If both tests (blood and saliva) were available in the future, which would you likely pick?				
Blood	6 (30)	–	11 (28)	–
Saliva	11 (55)	–	29 (72)	–
No preference, I like both	3 (15)	–	–	–
How much would you pay for this HIV test kit (oral or blood, respectively)?				
Range	10–200	10–300	0–300	–

Note: - indicates that the question was not asked or applicable, or that the response was zero

#### Phase 2a: Observation of both oral and blood-based test

During phase 2a, there were 20 participants, 10 young men (*median*_age_ = 19, interquartile range = 4) and 10 young women (*median*_age_ = 21, interquartile range = 2), who completed both a blood test and a saliva test. One participant was suffering from a dry mouth and could not produce enough saliva to collect a sample. Prior to study participation, 10% (*n* = 2) had never tested for HIV. Of the remaining participants, 65% (*n* = 13) reported testing for HIV in the past 6 months. The majority last tested at a clinic (78%, *n* = 14), with a smaller proportion testing at a non-government organization (NGO; 22%).

#### Phase 2b: Observation of either oral or blood-based test

During phase 2b, a total of 40 participants (50% female; *median*_age_ = 21) completed the HIV self-test of their choice. One participant, however, was unable to complete the saliva test, stating that he did not understand the instructions. Prior to study participation, 18% of participants had never been tested for HIV. Of the remaining participants, 33% reported testing for HIV in the past 6 months. The majority last tested at a clinic (61%) followed by a NGO (24%), followed by testing as part of previous research studies (12%) or private doctor (3%). Our results indicated that the most frequently observed errors occurred during the saliva test, as a number of participants failed to read the instructions (*n* = 9, 28%) or did not remove test tube cap before placing the tube in the stand provided in the saliva test kit (*n* = 7. 22%). In all cases, except one, participants and observers were concordant in their interpretation of the test result. In the only discordant observation, a participant interpreted the blood test result as negative and the observer interpreted it as positive. The observer noted that the participant did not see the second line indicating a positive result.

### HIVST acceptability

Overall, our results indicated that HIVST was acceptable to study participants in both phases 1 and 2. We identified several themes that elucidate positives and negatives about HIVST that are summarized in Table 3 and reported below.

**Table 3:** Overview of participants responses

Themes	Illustrative Quotations
HIVST acceptability	
Testing independently	*“It is good because [HIVST] could reduce the rates of HIV because [some people] are afraid to be tested by other people. It is a good idea because a person will test alone and get the result alone… It’s good because you test at home. If you test positive, no one will know. You will keep it to yourself until you get ready to disclose… It is good for me because my status will be known by me only, if it happens that other people know, it would be me who disclose.” (22-year-old male)*
	*“I am very close to my best friend, so I would like to be with her when I do the test”(20-year old male)*
	*“I cannot tell my best friend, because she has secrets she don’t tell me some of the things about her life, so why should I have to tell her or be with her, it means she don’t trust me so why should I have to trust her, so I will tell one of my brother from the church, I trust him so much.” (18-year-old female)*
User preferences	
Preferences for type of kit	*“I won’t tell my friends to use this test [blood based test] because they can feel the pains after using it. The saliva is better though the time to wait for the results is longer than this blood test. The main thing is getting the proper results as it is easy because you swab the saliva only.” (22-year-old male)*
	*“…We learned that HIV test can be easily be detected through blood or blood contamination. So now we are told that we can use saliva to test HIV. I don’t understand now. That is why people will prefer this blood test. It is the best.” (22-year-old male)*
Where to test	*Interviewer: “Do you think people of your age may have challenges in finding a place to do the test?”* *P14: “They will have difficulties”. (20 years old)* *I: “Where would they think they can do the test?”* *P18: “They can do it in their rooms.” (20 years old)* *P10: “At home sometimes we don’t have enough rooms we share our rooms. It would be better if there is a place like a voting station where we can do our test in private so that everyone will know their status alone. And there should be a dust bin there to throw rubbish in.” (18 years old)* (male focus group)
	*“Just create your own time, and to the test, use the little chance you get. Especially when you bath no you will take your time, people might think that you are bathing.” (21-year-old female)*
Materials accompanying the test kit	*“There should be a pamphlet in the kit that explains that has counselling message like written life goes on [even if you test positive].” (22-year-old male)*
Concerns about HIVST	
Validity of HIVST results	*“How sure are you about this kit? I don’t trust this kit. Why should I have to go back to the clinic and get tested again after using [the HIVST kit] and [to potentially] test positive? I cannot use [the HIVST]. I’d rather go to the clinic and use blood test, not [the blood HIVST].” (18-year-old male)*
	*“The saliva test is not easy to me because if I test and the results are invalid, will I then go to the clinic to test again? [The reason that] I collected the kit privately was because I wanted to test [on my own] and I don’t want the nurses to know my status or results. So, I [don’t like HIVST]… I want the type of test that I can do alone rather than having to go to the clinic [for confirmation].” (22-year-old, male)*
Lack of emotional support	*“…I don’t understand this type of test because if I can find out that I am HIV positive the time I do the test privately I will think of a lot of things because there won’t be anyone who will support me or at least [in the clinic] I get counselling that will [tell me] what I should do if I am HIV positive because I can think of committing suicide.” (25-year-old male)*
Costs of test	*“I think at the clinic and chemist [pharmacy] are good places because this test is also for health.” (18-year-old male)*
	*“I don’t agree about getting the kit at the chemist because not everyone will afford it because we have to buy the kit and mostly youth are unemployed, so they won’t get money to buy those test kit at the chemistry.” (24-year-old male)*

#### Testing independently

Focus group participants reported that HIVST would enable them to have more control over disclosing their status to others due to the increased confidentiality that the test would afford them. Similarly, participants from phase 2a cited the ability to complete the test independently as a primary incentive for engaging in HIVST, followed by the test being easy to use. Participants in phase 2a also reported more comfort with collecting saliva samples than they were collecting blood samples (Table 1). During phases 2a and 2b, almost all participants reported that it was very easy to collect the saliva sample (95 and 94%, respectively). Moreover, all participants were very confident that they could use the test independently and the overwhelming majority reported that, if they were to be tested for HIV again, they would prefer to use a HIVST kit at home (2a: 100%; 2b: 80%). Despite this, slightly more than half of the participants in 2a and some participants (19%) in 2b reported that they would like to have someone (e.g., a family member (*n* = 4) or sexual partner or a friend (*n* = 1), respectively), with them at home while engaging in HIVST. These findings are consistent with some of the responses from the focus groups, during which some participants expressed the importance of testing with either a close friend or relative to provide immediate access to social support. This sentiment, however, was not universal, as others expressed skepticism about testing with friends and suggested that the desire to test with others, whether friends or relatives, was dependent upon the quality of the relationship and interpersonal trust.

### User preferences

#### Preferences for type of kit

Focus group participants expressed preferences regarding the type of HIVST (i.e., saliva or blood-based) depending upon their key concerns about HIVST overall. Specifically, we found that participants who reported concerns about potential pain during HIVST showed a preference for the saliva-based test, while those most concerned about accuracy preferred the blood-based test. During phase 2a, most participants reported that they were very comfortable using the saliva test (84%), that it was painless (100%), and very easy to collect the sample and use the test (95%). During 2b most participants chose the saliva test (80%), reporting that they were very comfortable using the saliva test (77%), that it was painless (100%), and very easy to collect the sample (94%) and use the test (87%).

#### Where to test

Phase 1 focus group participants were asked to provide suggestions regarding places where a youth might feel comfortable engaging in HIVST. While most participants agreed that a bathroom or private bedroom would be the best place to take the test, a number of participants noted concerns. Participants, for example, suggested that these places might not be feasible for youth in their communities, as many share bathrooms and bedrooms with others and may not be afforded much privacy. As such, participants suggested that a degree of creativity (i.e., HIVST booths at convenient sites within the community) or pre-planning was essential to make time to test.

#### Materials accompanying the test kit

Participants were asked to share their thoughts and recommendations regarding the types of materials that should be included in the HIVST kits. For both test kits, participants in the phase 1 focus groups suggested that the kits include pre- and post-test counseling materials (e.g., pamphlets or resource lists), or provide a toll-free number where an individual is able to receive free counseling and encouragement in the event of a positive diagnosis. Additionally, focus group participants suggested that several materials should be included in the test kit, including wet and dry wipes to disinfect and dry one’s finger in preparation for the prick, gloves to avoid improperly handling the test kits, and a disposal bag to facilitate confidentiality.

### Concerns about HIVST

#### Validity of HIVST results

Despite the overall positive views about self-testing from participants in phase 1, some expressed concerns. Several focus group participants were troubled by the fact that test results could be invalid and return false positive or negative results. These participants believed that it would then be difficult to trust the results of HIVST, overall, and suggested that participants might even attribute positive test results to an unreliable HIVST kit rather than actually being HIV positive. Moreover, there were potential concerns about wasting time and/or money on a HIVST that could provide users with incorrect results or that requires a follow-up test at the clinic anyway. This seemed to be less of a concern for participants in phases 2a and 2b who actually had the opportunity to use the HIVST, as they unanimously reported that they trusted their HIVST test results. Participants did appear to have difficulties with using the blood-based kit, as 30% of participants received an invalid result compared to only 5% of those using the saliva test in phase 2a. Only one participant completing a saliva test in phase 2b received an invalid result and none who completed the blood-based test received an invalid result; however, significantly fewer individuals elected to use the blood kit.

#### Lack of emotional support

Though participants praised the HIVST for the privacy and confidentiality that it afforded users, a number of participants in phase 1 expressed concerns regarding a lack of emotional support should an individual be alone and test positive for HIV. Participants noted that traditional HIV testing in clinic settings gives patients access to immediate and often concurrent HIV counseling. There was uncertainty regarding how individuals would cope with a positive test in the absence of counseling and several believed that individuals might choose to end their lives as a result.

#### Costs of test

There were also concerns about the potential costs of a HIVST, as some participants worried about youths’ ability to purchase HIVST kits given the high rates of unemployment amongst youth.

## Discussion

We found that HIVST was both highly acceptable and preferable to traditional clinic-based testing among young, rural South Africans. This finding is consistent with previous research with South African youth in urban settings and adult men who have sex with men [19, 20]. Moreover, as in other studies, favorable attitudes toward HIVST were largely attributed to the tests’ ability to address critical barriers to HIVST, including: 1) confidentiality, 2) privacy, 3) convenience, and 4) control over disclosing one’s status to others [7, 14, 21]. In both the focus group discussions and observations of HIVST use, there was a strong preference for the saliva-based HIVST kit over the blood-based HIVST kit, as more participants reported that the saliva test was very easy to use when compared to the blood test. This finding differs from a previous study in rural South Africa among young men who have sex with men that showed a participant preference for the blood test [22]. It is notable that in phase 2b, almost a third of participants did not read the instructions prior to attempting to collect a saliva sample and some missed or reversed steps. This, however, did not appear to influence their ability to collect the sample. A number of youth reported that they would prefer to complete the test in the presence of a relative, friend, or sexual partner. These findings could have implications for the way in which HIVST kits are marketed, such that packaging HIVST kits in pairs could improve HIV testing uptake and also encourage those requiring additional support to test with a trusted companion (8).

Despite the positive views about HIVST, a number of focus group participants expressed noteworthy concerns. First, there were concerns about the lack of social support during HIVST, particularly for those who choose to test alone. Participants were especially concerned that a positive test could lead a person to behave irrationally and possibly attempt suicide. As suggested in previous research in South Africa and elsewhere, there are links between suicidal ideation and recent HIV diagnosis, as well as disparities in reports of suicidal ideation or past suicide attempts between people living with HIV/AIDS and their counterparts [23–27]. Providing individuals with access to both local and national resources for counseling and support services pre- and post-HIVST could reduce suicide risk within this population. Such resources could include 24-h hotlines, contact information for local mental health providers, or access to counseling applications for mobile devices that help individuals to make decisions regarding HIV testing using avatars suggesting that individual test in the presence of a trusted relative or friend [26]. In this way, we can provide further assurance that help is available to those who need it.

Second, some focus group participants expressed concerns about the validity of saliva-based HIV tests, believing that HIV was best detected using a blood-based test, which is consistent with previous research [20]. However, this did not diminish acceptability of self-testing within the groups. Moreover, there were concerns about the probability of receiving invalid test results, leading some participants to question the accuracy of HIVST. During phase 2a, more than a third of participants received invalid results using the blood-based test but only 5% received invalid results using the saliva-based test. It is possible that the manufacturer’s instructions or the design of the blood-based kit itself requires adjustment to improve one’s chances of obtaining valid results, as the instructions of the blood test required multiple steps. Test manufacturers could partner with communication and public health experts to develop HIVST instructions that are shorter, require fewer steps and are thus more user friendly. Relatedly, a number of focus group participants lamented the idea of engaging in a repeat HIV test within three months of HIVST for diagnostic verification purposes. It is notable that HIVST kits are considered to be screening devices and are therefore not intended to be diagnostic. For some participants, the necessity of being tested in the clinic rendered the HIVST less useful and suggested that it was better to be tested in the clinic the first time around to avoid unnecessary cost and demands on one’s time. Given that the primary incentive to engage in HIVST was related to increased confidentiality and independence, it will be critical to identify ways to continue to protect one’s privacy during repeat HIVST.

Participants suggested that it might be difficult for youth in their communities to find private places to engage in HIVST. While most suggested that a private bedroom or bathroom at home would be sufficient, some suggested that shared and often overcrowded living conditions could make that difficult. Youth, for example, might need to spend 20 min or more in the bathroom from start to finish, which some participants suggested could lead to suspicion. While there are no easy solutions for this particular challenge, most participants believed that youth would be able to find a place to test and would be less worried about this particular concern. Local clinics may be helpful in this regard. Task-shifting is one possible solution to concerns about privacy. For example, rather than being tested by clinic staff, lay counselors could be trained to show patients how to use the test and then allow them to test on their own in a small private room. Afterwards, a patient could indicate whether or not they would be interested in additional counseling. Alternatively, stakeholders could consider setting up HIVST booths at either clinics or in public locations (e.g., schools, churches, frequented community venues) that could provide people with a private place to test. Such an approach would give participants greater autonomy with regard to disclosing their status to staff, as well as in their decision to seek counseling or not. However, it might be important that such booths serve multiple purposes to maintain confidentiality and reduce stigma.

Lastly, there was concern about the potential cost of HIVST. Those at greatest risk for HIV infection may have significant challenges with regard to purchasing test kits. Many, for example, are unemployed and lack basic necessities. As a result, purchasing a HIVST kit may not be feasible. Participants suggested that HIVST kits be offered freely at local clinics or at pharmacies for reduced costs. There are, in fact, a number of initiatives in South Africa, as well as within other countries in sub-Saharan Africa, testing the feasibility of wide-scale distribution of HIVST kits [28, 29].

For those who choose to engage in HIVST, focus group participants suggested that materials (e.g., dry wipes, glove us, and a disposal bag) should be included in the test kit to facilitate hygienic handling and proper kit disposal. In rural, underserved settings, such materials may not be readily available in one’s home. Therefore, including them with the kit could not only relieve potential burden, but could also help to ensure that participants do not contaminate the sample, which could increase the likelihood of valid results. Despite the reservations expressed during focus groups, acceptability remained high. Moreover, among participants who engaged in HIVST during the pilots, the feedback was overwhelmingly positive and most reported that they would prefer to engage in HIVST in the future. It is likely that direct experience with the test kits helps youth to overcome initials reservations that they may have about their ability to engage in HIVST. As such, supervised practice and instruction prior to testing independently might be required for some youth, particularly those interested in the blood-based test.

There are a number of potential limitations associated with the current study. First, we are limited by a small sample size; as a result, we cannot guarantee that our results are generalizable beyond study participants. Second, participants were conveniently sampled; therefore, views expressed during focus groups and the direct observation studies may not be generalizable to all young people. Finally, we did not confirm the HIV status of focus group participants, which could potentially shape views regarding HIVST.

## Conclusions

Our results indicated that HIVST was highly acceptable to young, South African male and female participants residing in a rural community. However, a number of concerns were raised that require attention in order to improve HIVST uptake within this population. Participants overwhelmingly preferred the saliva-based HIVST kit despite some believing that the blood-based test was more accurate. HIVST has the potential to address key barriers to HIV-testing in community-based settings; however, key measures need to be taken to ensure that end users are provided with necessary support resources to protect against unintended negative outcomes, such as suicidal behavior. It is therefore critical that researchers, product developers, and end users partner to determine best approaches to HIV testing within specific populations.

## Additional file

Additional file 1: List of questions guiding the semi-structured interviews (DOCX 24 kb)

## Abbreviations

AIDS: Acquired Immune deficiency syndrome
HDSS: Agincourt Health and Socio-demographic Surveillance Site
HIV: Human immunodeficiency virus
HIVST: HIV self-testing
M: Mean
NGO: Non-government organization
