# Supplementary material for: HIV self-testing: South African young adults’ recommendations for ease of use, test kit contents, accessibility, and supportive resources
Source: BMC Public Health. 2019 Jan 29;19:123. doi: 10.1186/s12889-019-6402-4 (PMC6352366; doi:10.1186/s12889-019-6402-4)
Supplement: Supplementary file 1 — List of questions guiding the semi-structured interviews (DOCX 24 kb) [file 12889_2019_6402_MOESM1_ESM.docx]

**Innovations in HIV testing to enhance care for young women and their peers and partners: A pilot acceptability and feasibility study**

**INTRODUCTION SCRIPT**

**Current testing experiences and barriers**

Let’s begin by asking you a few questions about HIV in your community.

- How do your friends or peers learn their HIV status – or whether they have HIV or not?
- What are people your age in your community saying about HIV testing?

**DEMONSTRATION OF TEST KITS:**

I will now show you two different types of home test kits. First, I will show you the test that uses saliva from your mouth and then ask a few questions about it.

*(Demonstrate the Oral Test Kit without actually doing the test: Ask if people if they would like to see the testing procedure demonstrated again or if there are any questions)*

**Reactions to home testing following demonstration**

This is how the test would work – and people would do this themselves at home.

- Now that I have shown you how to use the saliva test,
  - What do you think about the saliva test?
  - What would your friends think about using the saliva test at home?
  - What do you like about the test that uses the saliva?
  - What do you not like about the test that uses saliva?
  - If you thought that you needed to be tested for HIV, would you be willing to use the saliva test? Why/why not?

Now, I will show you the home test kit that uses blood rather than saliva. I will demonstrate how it’s used.

(*Demonstrate with kit as the following steps are explained, without actually drawing any blood)*

*(Ask people if they’d like to see testing procedure demonstrated again or if there are any questions).*

**Reactions to home testing following demonstration**

This is how the test would work – and people would do this themselves at home.

- Now that I have shown you how to use the blood test,
  - What do you think about the blood test?
  - What would your friends think about using the blood test at home?
  - What do you like about the blood-based test?
  - What do you not like about the blood-based test?
- If you thought that you needed to be tested for HIV, would you be willing to use the blood test? Why/why not?
- Would you recommend it to someone your age who needed to be tested? Why/why not?
- Do you have a preference between the two tests that we demonstrated today? Why? Why not?

For either the saliva or blood-based test:

- - How would people your age feel about taking one of these HIV tests at home?
- Do you think your people your age would be able to do these tests at home by themselves? Why? or Why not?
- What would determine whether people your age tested alone?
- In what situations do you think your friends would use one of these tests?
- How often do you think people would use these tests?
- What kinds of materials would make these tests easier to use at home?

**Proposed study**

We are planning to conduct a study in this area where we give young women self-test kits to take home to use. We also will give them kits to give to their friends and partners/boyfriends.

- If you were asked to participate in this study, would you? Why or why not?
- What would be the best way to recruit young women to participate in this study?
- Would a young woman be willing to give a test kit to a friend? Why or why not?
- Would a young woman be willing to give a test kit to a sex partner/boyfriend/girlfriend? Why or why not?
- Tell me what you think a sex partner/boyfriend might say if a young woman invited them to participate in this study?
- In this study, we would need to get in touch with you about your experience after we give you the test kit. What would be the best way?
- What would be the best way for us to get in touch with the friends or sex partners/boyfriend that you give test kits to so we can find out about their experiences with the test?

**Recommendations for Ease of Use / Tools / Support**

- If these test kits were available today – what materials should be included inside of the kit or box that you would take home?
- If you were going to use a test kit, what other resources would you like to have available?
- If you needed support, before, during, or after taking the test, who would you reach out to?
- If these tests where available today, where would young people want to get them?

**Interpreting Positive Test Results**

Now I want to talk about what it means to get a positive test result. What we are going to discuss regarding a positive test is true for both of the tests that you saw demonstrated today. It is very important to know that a positive test with a self-test kit does NOT always mean that you are infected with HIV. Some people who do NOT have HIV incorrectly test positive using these tests, which is why follow-up testing is extremely important to confirm HIV status.

- Imagine for a moment that a friend tests positive at home. That experience could be difficult so we need to know what we can do to help with this experience. What kind of support should be available for this friend if they tested positive at home?
- What kind of support would your friend need to help them get follow-up HIV testing to determine if they are actually infected with HIV?
- What kind of support would *you* want to receive if you had a positive test at home?
- What kind of support/resources would you need to help you get follow-up HIV testing?

**Interpreting Negative Test Results**

Now, imagine for a moment that a friend uses the test and gets a negative test result. What we are going to discuss regarding a negative test is true for both of the tests that you saw demonstrated today.

If you have had a recent exposure to HIV in the past 3 months, HIV may not yet be detectable in your blood. This means that people who are just recently infected with HIV may have a negative test result even though they do have HIV.

**Closing remarks**

- Given all that we have said today, what your thoughts are now about HIV self-testing?
- What else is important about HIV self-testing, with either the blood-based or the oral test – including potential benefits or concerns – that we haven’t yet discussed today?

Thank you for your participation.
